# Supplementary material for: Topological regression as an interpretable and efficient tool for quantitative structure-activity relationship modeling
Source: Nat Commun. 2024 Jun 13;15:5072. doi: 10.1038/s41467-024-49372-0 (PMC11176398; doi:10.1038/s41467-024-49372-0)
Supplement: Supplementary file 1 — Supplementary Information [file 41467_2024_49372_MOESM1_ESM.pdf]

# Supplementary Materials for Topological Regression in Quantitative Structure-Activity Relationship Modeling

Ruibo Zhang[1]†, Daniel Nolte[1]†, Cesar Sanchez-Villalobos[1], Souparno Ghosh[2]∗, Ranadip Pal[1]∗

[1] Dept. Electrical and Computer Engineering, Texas Tech University
[2] Department of Statistics, University of Nebraska - Lincoln
∗Corresponding author(s).
†These authors contributed equally.

## 1 Molecular descriptors/fingerprints performance comparison on ChEMBL datasets

The choice of descriptors or FPs is critical to the performance of QSAR predictions. We compared four commonly used descriptors/FPs that are considered to have superior performance, plus the default parameters of ChemProp. Four descriptors/FPs are compared by their RF prediction scores. As we have 530 sub-datasets, the performances are compared by their distribution shown in kernel density estimate (KDE) plots in Fig. S1.

By comparing the mean and distributions of the descriptors, we show that ECFP4 has superior prediction performance overall. The ranking relationship can be written from superior to inferior as $ECFP4 > Mordred \approx RDKit \approx ChemProp > TF3P$. The results on other models show a similar conclusion, which are provided in Fig. S2 and S3. In the scaffold splitting setup and under the Spearman metric, the advances of ECFP4 arenot as significant, the rankings can be written as $ChemProp \approx ECFP4 \approx Mordred > RDKit > TF3P$. In general, ECFP4 shows higher Spearman and $R^2$ scores compared to the other descriptors. Therefore, we chose to use the ECFP4 fingerprints for the FP-based models and ECFP4 distances for TR throughout the paper.

In the KDE plots, we selected random forest (RF) as the standard to compare the predictive power of different chemical descriptors/fingerprints. Even though RF gives robust performance and fair comparison of the descriptors, a thorough comparison using other models is still desired. Next, we compared the descriptors / FPs with RF, ridge regression, support vector regression (SVR), and in both random cross-validation and scaffold split scenarios. Results are provided in box plots shown in Fig. S2 and S3. Among all compared descriptors, ECFP4 shows robust and better results compared to the others. These results provide a guideline for selecting descriptors and regression models for similar tasks.

## 2 The role of response landscape in QSAR model prediction

Topological regression (TR) uses distance mapping to account for rapid changes in the response landscape. Intuitively, TR accommodates cliffs in landscapes, making it particularly effective for datasets with rugged response landscapes. We demonstrate this characteristic by plotting model performance differences against the prevalence of "cliffs" in dataset responses.

We use the structure-activity landscape index (SALI) to measure the prevalence of cliffs in a dataset [1]:

$$SALI_{i,j} = \frac{|A_i - A_j|}{1 - sim(i,j)} \tag{1}$$

The SALI between a pair of molecules is calculated as the difference in activity divided by their dissimilarity in structure. In this study, chemical similarities are determined using ECFP4-Tanimoto coefficients (TC). When two molecules have identical ECFP4 fingerprints, the SALI is defined as 0. For a dataset comprising $N$ drugs, the median SALI value of all $(N-1)^2$ molecule pairs is used as the metric.

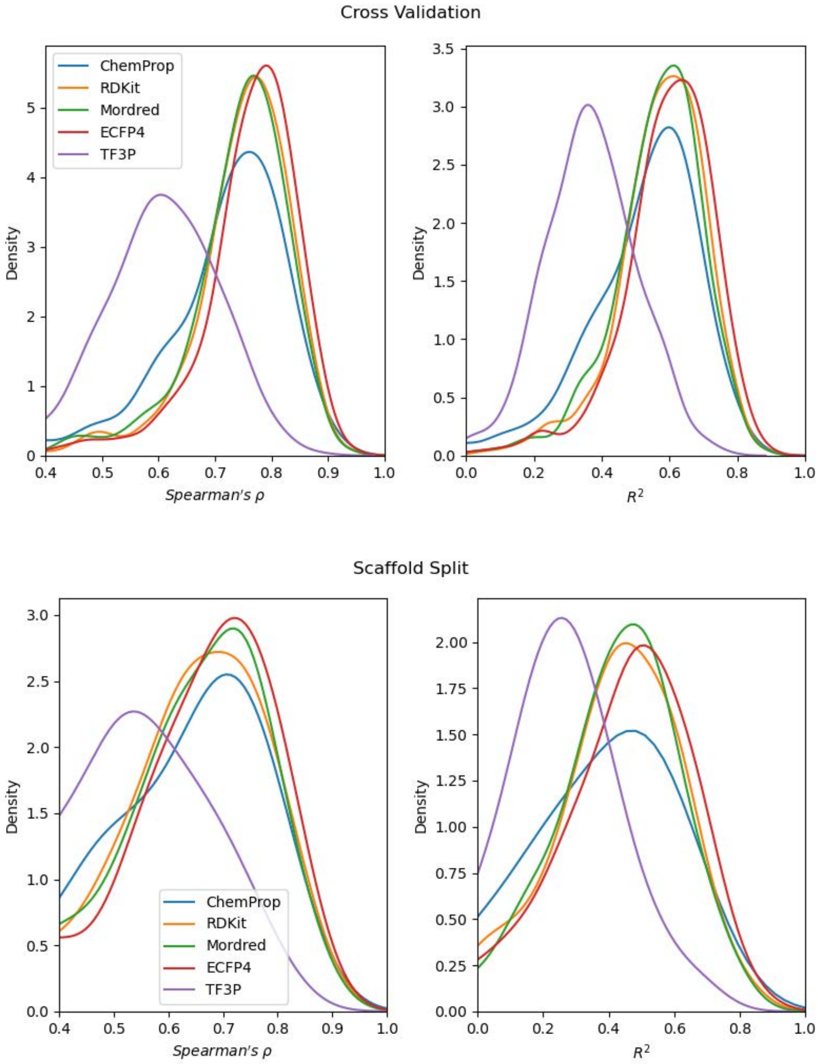


Figure S1: **Descriptor/Fingerprint comparison on 530 ChEMBL bioactivity datasets under random cross-validation and scaffold split setups.** The distribution of all performance scores is shown by kernel density estimate plots with the same bandwidth, and higher scores mean better performance. All descriptors/Fingerprints except ChemProp were used with Random Forest as a subsequent prediction model. ECFP4 shows superior scores than other methods.

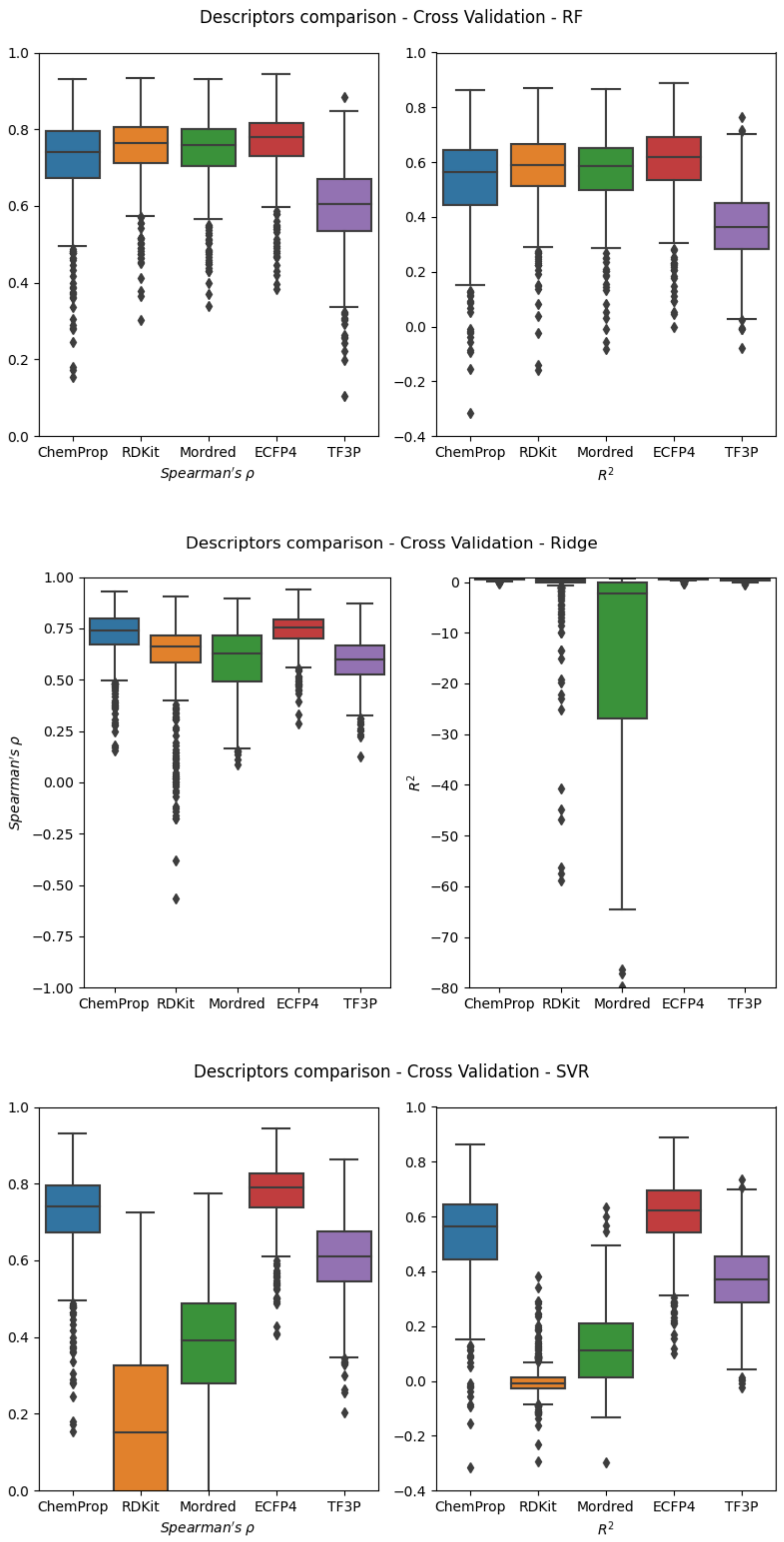


Figure S2: **Descriptor/Fingerprint comparison on all 530 ChEMBL bioactivity datasets under random cross-validation.** Descriptors are compared with Random Forest (RF), ridge regression, and Support Vector Regression (SVR). The ECFP4 descriptors show a higher correlation on the three analyzed models. The box plots show the median (central line), the interquartile range (upper and lower limits of the box), and the 5% and 95% limits (whiskers), as well as the outliers on $n = 530$ ChEMBL bioactivity datasets.

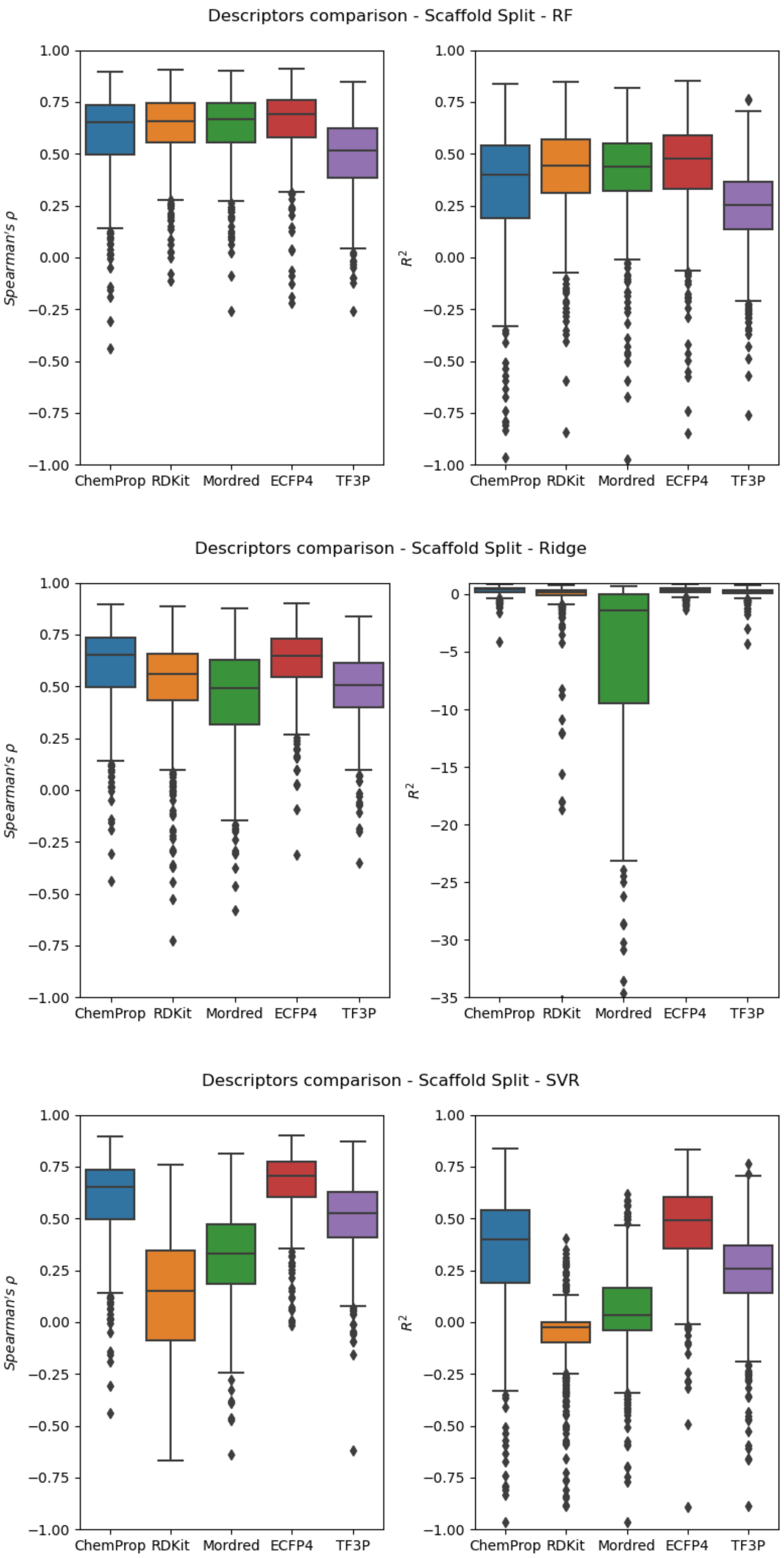


Figure S3: **Descriptor/Fingerprint comparison on all 530 ChEMBL bioactivity datasets under scaffold split.** Descriptors are compared with Random Forest (RF), ridge regression, and Support Vector Regression (SVR). The ECFP4 descriptors show a higher correlation on the three analyzed models. The box plots show the median (central line), the interquartile range (upper and lower limits of the box), and the 5% and 95% limits (whiskers), as well as the outliers on $n = 530$ ChEMBL bioactivity datasets.

We plot the difference of $R^2$ scores of TR vs. plain RBF KDE, RF, and SVR, i.e. $R^2_{TR} - R^2_{othermodel}$, against the median SALI of each dataset. As shown in Fig. S4, TR shows more advantage in datasets with higher SALI over the other three models. All results are obtained from random cross-validation.

## 3 Assessing the adequacy of TR

TR utilizes multivariate general linear models for the extraction and modeling of the $W'_{i,j}s$. This allows incorporating tests for the goodness-of-fit, in comparison to other methods like RF, MLKR, TCNN, and ChemProp which all resort to unbounded comparative measures (NRMSE, MAE, etc.) for assessing the model performance. To evaluate the adequacy of TR, we computed the training R-sq values of the linear model in equation 3 which connects the $W_{i,j}$'s with $d^2_{i,j}$'s. The average training R-sq across all datasets was 0.8396 for the scaffold split and 0.8349 for the CV split. To expand this for predictive adequacy, we computed the 95% prediction interval for each test point[2]. Figures S6 and S5 depict the 95% prediction intervals for datasets targeting Coagulation factor XIII (CHEMBL4530) and Phospholipase D2 (CHEMBL2734). The average coverage of the 95% prediction intervals across all 5 CV folds and 530 datasets was calculated to be 94.3%. Therefore, the adequacy of TR, and specifically the model specified in equation 3, is justified for prediction purposes.

## 4 The effect of sample size for the competing methods

To evaluate the effect of sample size for the various models, we discretized the target datasets into 10 quantiles based on sample size and took the average scaffold split NRMSE for each quantile for each competing method. Figure S7 displays the average binned NMRSE across sample size for the various modeling methods under the scaffold split. As indicated by the graph, TR Ensemble outperforms TCNN with augmentation on the smaller sample size datasets while the performance gap closes as the dataset size increases. In total, TR Ensemble outperforms TCNN with augmentation on 293 of the 530 total target datasets and 424 of the 530 datasets when augmentation is not performed.

## 5 Additional TR interpretation Figures

In the main manuscript, we highlighted how TR can be used for interpretability of model predictions and as a potential tool for chemists to traverse the chemical space. To further show this behavior across other target datasets, we have supplied additional interpretability figures on various target datasets. In Figs S8 and S9, synonymous to Figure 5 of the main manuscript, we offer a visual comparison of the predicted neighbors of TR, KNN, and MLKR as well. In Fig. S8 we show the results for the target molecule CHEMBL4211876 (pChEMBL= 7.04) extracted from the dataset targeting Cytochrome P450 1A1, also referred to as CHEMBL2231. Once again, the target is located at the boundary of a high-activity region. Standard KNN can only find 3 nearest neighbors in the low activity region and hence underpredicts the response of the target. But, both MLKR and TR generate cross-scaffold predictions, offering considerable improvement over KNN predictions. In Fig. S9, we show the results for the dataset corresponding to the target Mitogen-activated protein kinase 12 with target molecule CHEMBL3730758. Interestingly, KNN finds all three neighbors in the high activity region and overpredicts the response of the target. But, both MLKR and TR offer predictions that are closer to the true value.

In figure S10 we have provided additional KNN-graphs for the dataset targeting Coagulation factor XIII, or CHEMBL4530, with training prediction clusters in the left column, test prediction clusters in the middle column, and the right column depicting 6 molecules from the most active cluster. Similar to Figure 6 in the manuscript, we see that TR results in much smoother and more homogeneous clusters. Finally, using the same target dataset, Figure S11 depicts a lead optimization pathway from the most active TR training cluster, the same as Figure 8 in the main manuscript. As seen, a chemist could easily traverse the different paths of the different clusters for actionable information to guide future designs.

## References

[1] Rajarshi Guha. Exploring structure–activity data using the landscape paradigm. *Wiley Interdisciplinary Reviews: Computational Molecular Science*, 2(6):829–841, 2012.

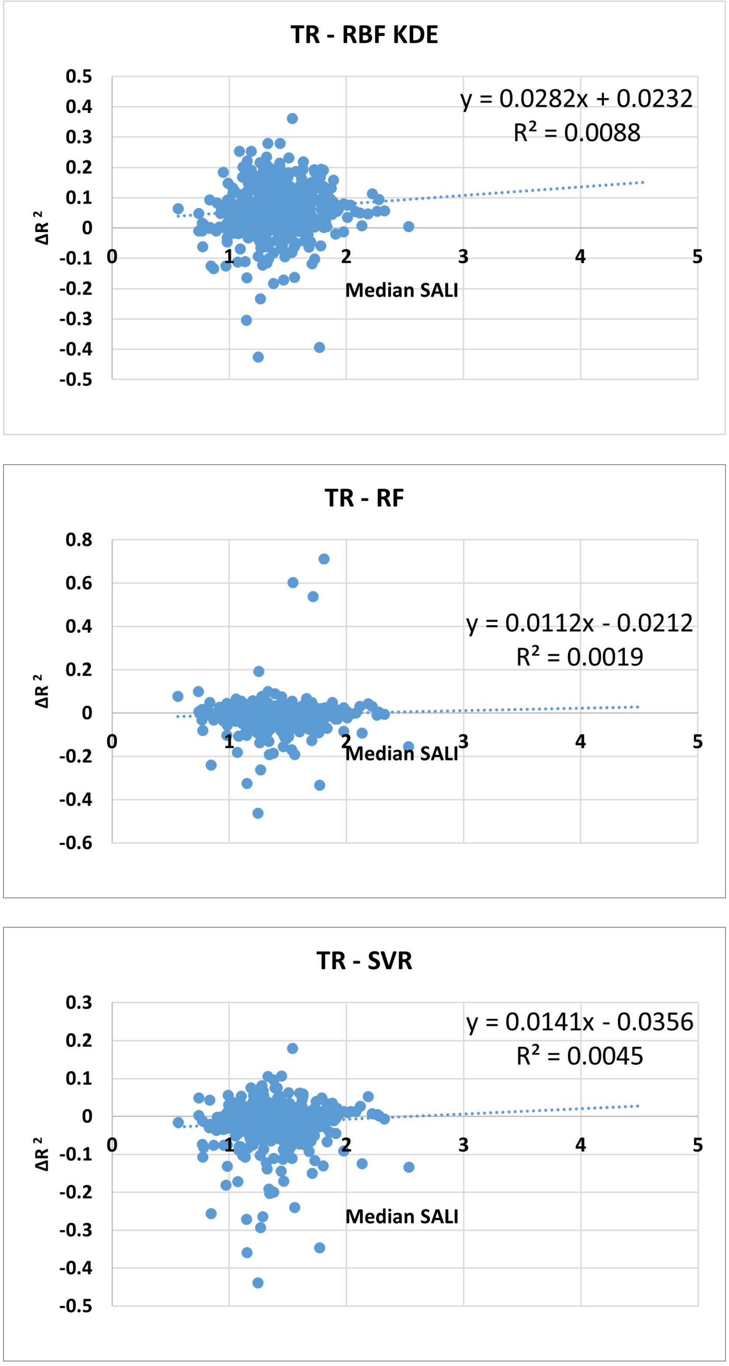


Figure S4: **Visualizing the difference in $R^2$ scores of TR vs. other models against the median SALI of the dataset.** The difference in $R^2$ scores ($\Delta R^2$) between Topological Regression (TR) vs plain Radial Basis Function Kernel Density Estimation (RBF KDE), Random Forest (RF), and Support Vector Regression (SVR) were plotted against the median structure-activity landscape index (SALI) of each dataset. The positive slope of the trend lines indicates that TR is more advantageous in datasets with higher SALI over the other three models.

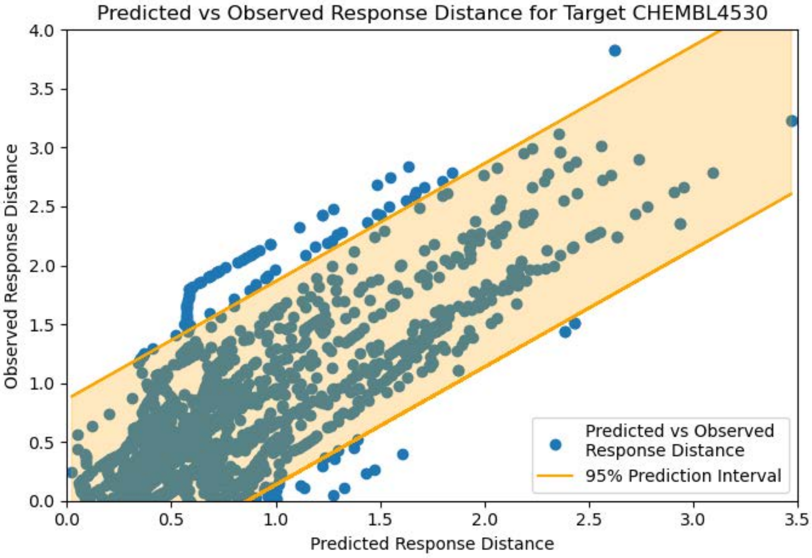


Figure S5: **Scatter plot of predicted vs observed response distances of target CHEMBL4530 with 95% prediction interval.** The prediction intervals approximately achieve nominal coverage justifying the adequacy of Topological Regression.

[2] Richard Arnold Johnson, Dean W Wichern, et al. Applied multivariate statistical analysis. 2002.

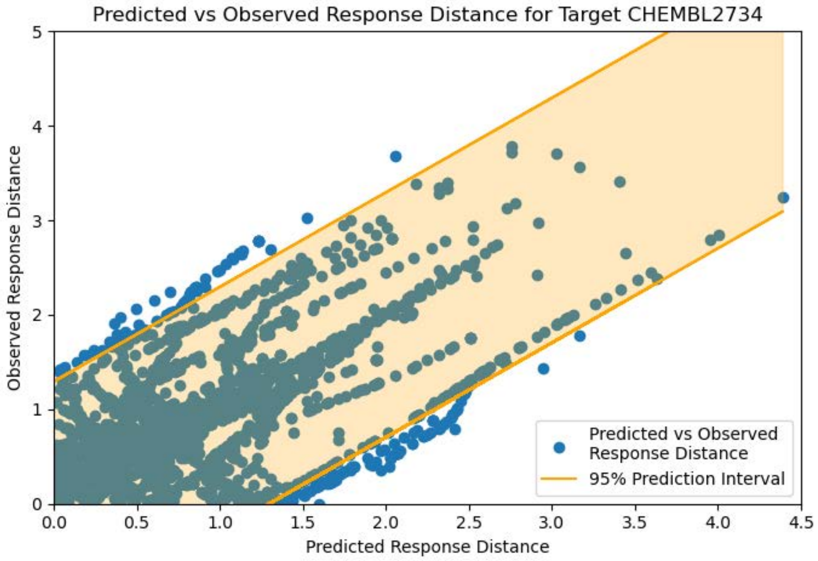


Figure S6: **Scatter plot of predicted vs observed response distances of target CHEMBL2734 with 95% prediction interval.** The prediction intervals approximately achieve nominal coverage justifying the adequacy of Topological Regression.

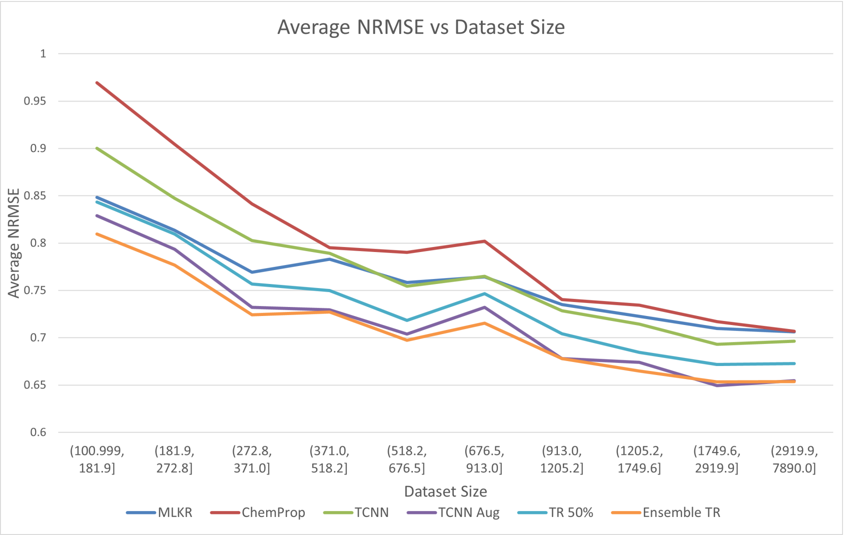


Figure S7: **Visualizing the effect of sample size for the competing methods.** The target datasets were binned into 10 quantiles based on sample size and the average Normalized Root Mean Squared Error (NRMSE) for each competing method was calculated and displayed for the scaffold split.

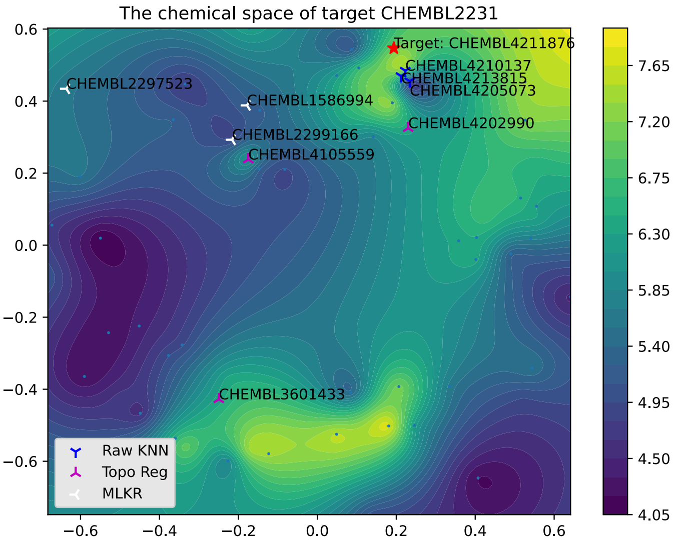


Figure S8: **Comparative analysis of the neighbors found by KNN, MLKR, and TR for CHEMBL4211876.** Visualizing three nearest neighbor configurations for the target molecule CHEMBL4211876 (pChEMBL=7.04) in a 2-D multi-dimensional scaling space extracted from the dataset consisting of molecules targeting Cytochrome P450 1A1 with the color representing the pChEMBL values. K-Nearest Neighbors (KNN) nearest neighbors are close only in the chemical space (KNN prediction = 4.85), whereas, Metric Learning for Kernel Regression (MLKR) (MLKR prediction = 5.21) and Topological Regression (TR) (TR prediction = 6.05) finds nearest neighbors in the response space.

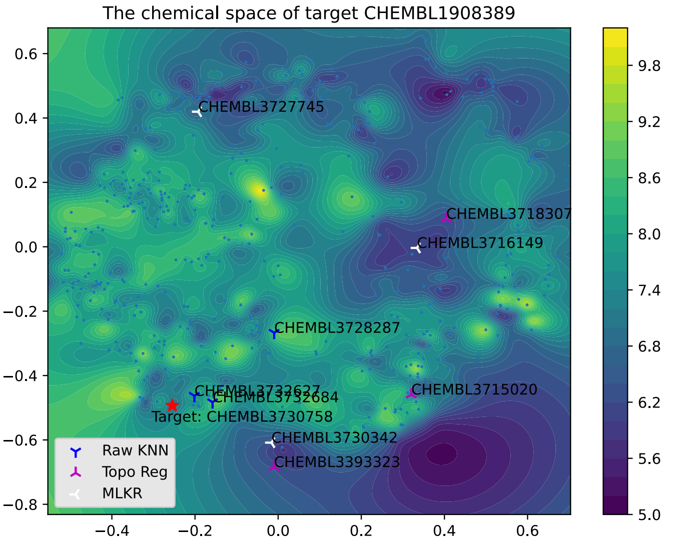


Figure S9: **Comparative analysis of the neighbors found by KNN, MLKR, and TR for CHEMBL3730758.** Visualizing three nearest neighbor configurations for the target molecule CHEMBL3730758 (pChEMBL = 7.01) in a 2-D multi-dimensional scaling space extracted from the dataset consisting of molecules targeting Mitogen-activated protein kinase 12 with the color representing the pChEMBL values. K-Nearest Neighbors (KNN) overpredicts pChEMBL value (KNN prediction = 8.71). Metric Learning for Kernel Regression (MLKR) (MLKR prediction = 6.19) and Topological Regression (TR) (MLKR prediction = 6.33) predictions are more tempered.

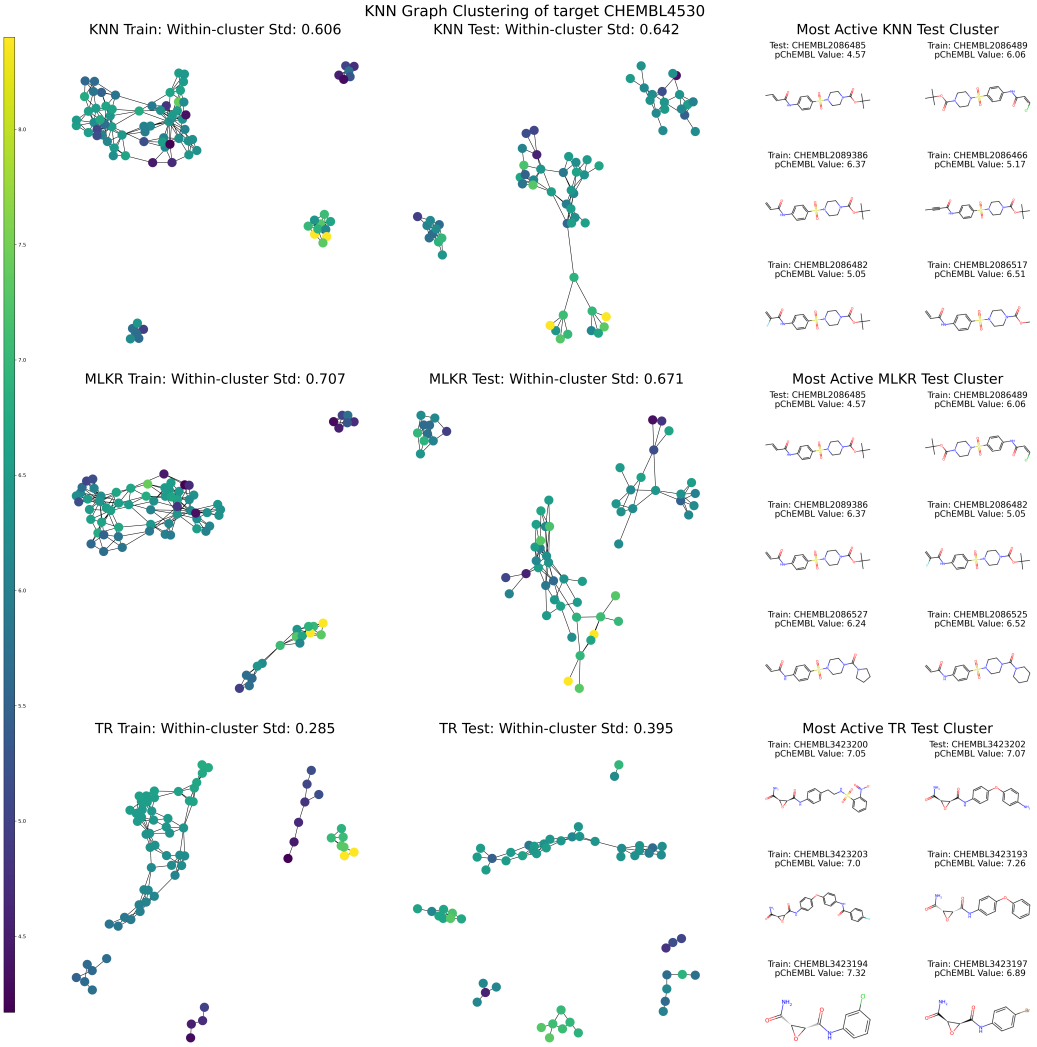


Figure S10: **Training and test k-Neared neighbor Graph Clusters of ChEMBL4530.** The left column contains the clustered training predictions, the middle contains the clustered test predictions, and the right column contains 6 molecules from the most active cluster.

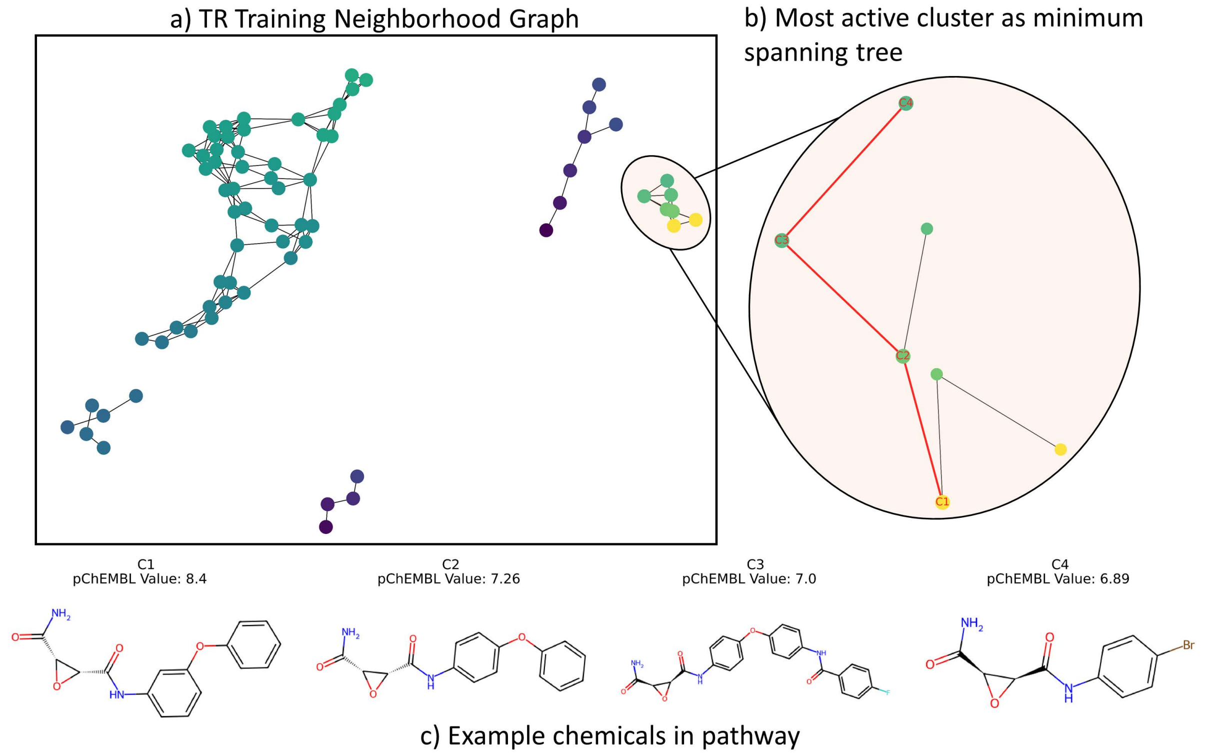


Figure S11: **Optimization pathway visualization in the most active training cluster of target CHEMBL4530.** a) depicts the training neighborhood graph obtained from TR predictions, b) depicts the minimum spanning tree of the most active cluster with a minimum path connecting the most active and least active molecules, c) 4 example molecules showing the lead optimization pathway.
